# Clinical characteristics, antimicrobial resistance, and mortality of neonatal bloodstream infections in Northern Tanzania, 2022–2023

**DOI:** 10.1371/journal.pone.0319816

**Published:** 2025-03-25

**Authors:** Ganga S. Moorthy, Matthew P. Rubach, Anna Sechu, Ronald Mbwasi, Nyemo Peter, Ibukunoluwa C. Kalu, John A. Crump, Dorothy E. Dow, Blandina T. Mmbaga, Aisa Shayo

**Affiliations:** 1 Division of Pediatric Infectious Diseases, Duke University Medical Center, Durham, North Carolina, United States of America; 2 Duke Global Health Institute, Duke University, Durham, North Carolina, United States of America; 3 Division of Infectious Diseases and International Health, Duke University Medical Center, Durham, North Carolina, United States of America; 4 Kilimanjaro Christian Medical University College, Tumaini University, Moshi, Tanzania; 5 Programme in Emerging Infectious Diseases, Duke-National University of Singapore Medical School, SingaporeSingapore; 6 Kilimanjaro Christian Medical Centre, Moshi, Tanzania; 7 Centre for International Health, University of Otago, Dunedin, New Zealand; 8 Kilimanjaro Clinical Research Institute, Moshi, Tanzania; University of Bologna / Romagna Local Health Authority, ITALY

## Abstract

Neonatal bloodstream infections (BSI) make a substantial contribution to morbidity and mortality in low- and middle-income countries (LMICs), but data on the epidemiology and antimicrobial resistance (AMR) in Tanzania are limited. We describe the prevalence, resistance patterns, and associated factors of neonatal BSI at the Kilimanjaro Christian Medical Centre (KCMC), a large referral hospital in northern Tanzania. We conducted a prospective, observational study involving infants aged 0–60 days with perinatal risk factors or clinical signs of sepsis. Aerobic blood cultures were obtained at enrollment and monitored using a continuously monitored blood culture instrument. Antimicrobial susceptibility testing was performed using standard phenotypic methods. Vital status was obtained on days 2, 7, and 28 post-enrollment. BSI was defined as the isolation of established neonatal pathogens, including yeast and coagulase-negative *Staphylococcus* spp. (CoNS). Early-onset BSI occurred on day of life (DOL) 0-2, while late-onset BSI occurred on DOL 3 or later. Among 236 enrolled infants, blood culture was obtained in 233. BSI occurred in 106 (45.5%) of 233 infants, 50 (47.2%) were early-onset, and 56 (52.8%) were late-onset BSI. The isolated pathogens included 58 (54.7%) Gram-positive bacteria, 40 (37.7%) Gram-negative bacteria, and 8 (7.5%) yeast. CoNS (n = 55, 51.9%) and *Klebsiella pneumoniae* (n = 35, 33.0%) were the most common pathogens. Notably, all *K. pneumoniae* isolates were extended-spectrum beta-lactamase producers, resistant to ampicillin and ceftriaxone. Among the 56 infants who died, 29 (51.8%) had BSI; 11 (19.6%) infants with EO-BSI, and 18 (32.1%) with LO-BSI. Infants requiring respiratory support at admission had a 1.89-fold increased adjusted odds of BSI (95% CI, 1.05-3.44). We found high prevalence of neonatal BSI due to bacteria with a high prevalence of AMR, and BSI was associated with high mortality. There is an urgent need for effective preventive, diagnostic, and therapeutic interventions to address BSI among hospitalized infants in northern Tanzania.

## Introduction

Neonatal sepsis causes 200,000 deaths worldwide annually, with the highest mortality in sub-Saharan Africa [1,2]. Despite progress in reducing childhood mortality and improving access to facility-based delivery and newborn care, many low- and middle-income countries (LMIC) will not meet the 2030 Sustainable Development Goal to reduce neonatal mortality to at least 12 per 1,000 live births [2]. Risk factors for neonatal sepsis include low birthweight, prematurity, premature rupture of membranes, intrapartum complications, and low socioeconomic status [3]. Diagnosing neonatal sepsis proves challenging as infants often show nonspecific symptoms such as lethargy and temperature variability. Pathogens causing neonatal bloodstream infection (BSI), a type of neonatal sepsis identified through blood culture, can originate from either vertical or horizontal transmission, depending on the timing of infection [4]. Early-onset BSI (EO-BSI), typically defined as occurring within the 0-2 days of life, is commonly attributed to vertical transmission from *in utero* infection or maternal flora at or around the time of delivery. Late-onset BSI (LO-BSI) can result from vertical transmission as well as horizontal or nosocomial transmission from the infant’s environment [5]. Although treating BSI based on blood culture data and local epidemiology is ideal, limited diagnostic capacity in resource-limited areas often make this approach unfeasible [3].

For neonatal care units in LMICs, Gram-negative bacteria (GNB) such as *Klebsiella* spp., *Escherichia coli*, and *Acinetobacter baumannii* are common pathogens in LMIC neonatal care units [6]. *K. pneumoniae* is the leading cause of BSI, responsible for 10% of neonatal deaths in LMICs [6–9]. The World Health Organization (WHO) recommends using a narrow-spectrum beta-lactam and gentamicin as first-line and third generation cephalosporins as second-line empiric treatments for neonatal sepsis [10]. However, widespread antimicrobial resistance (AMR) increasingly renders these empiric treatments ineffective [6,11]. In sub-Saharan Africa, the prevalence of resistance to first-line therapies can reach 50-90%, and AMR contributes to an estimated 30% of global neonatal sepsis deaths [6,9,12,13].

The epidemiology and antimicrobial resistance patterns of BSI in Tanzania are understudied. To address this, we describe the prevalence, resistance patterns, and associated factors of neonatal BSI at a large referral hospital in northern Tanzania, aiming to inform treatment algorithms and to guide future antimicrobial stewardship and infection prevention efforts.

## Methods

### Study aims

The primary aim of this study was to describe the prevalence of culture-confirmed BSI among very young infants admitted to the neonatal ward at a tertiary referral hospital in northern Tanzania. Secondary aims included: (1) describing antimicrobial resistance patterns of BSI isolates, (2) determining all-cause and BSI-associated mortality of enrolled infants, and (3) identifying infant and maternal factors associated with BSI.

### Setting

Kilimanjaro Christian Medical Centre (KCMC) is a 630-bed, zonal referral hospital located in Moshi, Tanzania. Moshi had a population of > 221,000 in 2022 and is at an elevation of approximately 890 meters above sea level [14]. KCMC serves the Regions of Kilimanjaro, Tanga, Arusha, Manyara, Dodoma, and Singida in northern Tanzania. At the time of this study, infants ≤  30 days of age born inside and outside of KCMC were admitted to a 79-bed neonatal ward. In 2023, 1,291 infants were admitted to the ward with an average of 108 admissions each month. Among infants admitted to the unit from January through December 2023, 68 blood cultures were ordered per 100 infants. The annual all-cause infant mortality was 22% in 2023. The ward has an intensive care unit, a high acuity unit, an area for infants with suspected or documented infection, a well-baby area, and a Kangaroo-Mother Care area. Pregnant women in Tanzania do not routinely receive Group B *Streptococcus* (GBS) testing or intrapartum GBS prophylaxis. The Tanzanian Ministry of Health Antenatal Guidelines recommend that pregnant women receive HIV testing and treatment, syphilis testing and treatment, and 3 doses of sulfadoxine-pyrimethamine for intermittent preventative treatment of malaria in antenatal clinics [15]. Clinicians in the ward generally follow WHO recommendations for neonatal sepsis management. Typically, a narrow-spectrum beta-lactam and gentamicin are used as first-line and ceftriaxone as second-line empiric treatment for neonatal sepsis [10]. However empiric antibacterial choice may vary based on individual patient factors, clinical acuity, and clinician’s practice patterns. Central access such as umbilical catheters or peripherally inserted central catheters are not used at KCMC, and infants receive medications and fluids via peripheral intravenous catheters or nasogastric and orogastric tubes. Respiratory support can be provided using nasal cannula, continuous positive airway pressure (CPAP), and ventilators.

### Study participants

We conducted a prospective, observational study of infants admitted to the neonatal ward in KCMC from 29 September 2022 through 28 April 2023. Infants aged 0-60 days with perinatal risk factors or clinical signs or symptoms of sepsis were eligible for enrollment. While the neonatal ward admitted infants ≤  30 days old, we allowed the inclusion of infants up to 60 days due to some having longer hospital stays. Study research nurses identified eligible new admissions and admitted patients at risk for sepsis by reviewing the admission log and consulting with physicians and nurses daily. Study staff screened and enrolled patients on Monday to Friday from 8:00 AM to 4:00 PM. Inclusion criteria were based upon WHO criteria and the modified criteria from the Burden of Antibiotic Resistance in Neonates from Developing Societies (BARNARDS) study (**Table 1**) [10,16]. Patients were excluded if a blood culture was obtained at KCMC within the last 48 hours, the parent or guardian was not present to provide consent, or if the parent or guardian did not speak Kiswahili or English.

**Table 1:** Enrollment criteria by age for neonates and infants with suspected sepsis in the neonatal ward of Kilimanjaro Christian Medical Centre from September 2022 through April 2023.

Infants 0–28 days (neonatal period)	
Prenatal Risk Factors at Birth	Signs/symptoms
Premature rupture of membranes ≥ 18 hours Maternal fever (axillary temp > 37.5ºC) at time of delivery Perinatal asphyxia (Apgar score ≤ 5 at 5 minutes) Foul smelling amniotic fluid Very low birth weight ( < 1,500g) Home birth	Difficulty or refusal to suckle Excessive crying, irritability Drowsiness, slow reaction times, hypotonia, coma Bulging fontanelle Convulsions Periumbilical erythema Respiratory rate > 60/min Severe chest indrawing Apnea ( > 15s) or bradypnea (respiratory rate < 20/min) Hypothermia (<35.5ºC) or fever (axillary temperature > 37.5ºC) Purulent discharge from the eyes • Crepitations/crackles on pulmonary auscultation Numerous skin pustules
**Criteria for inclusion: 1 risk factor OR 1 sign/symptom**	
**Infants 29–60 days (newborn period)**	
**Category A signs/symptoms**	**Category B signs/symptoms**
Purulent otorrhea Drowsiness, slow reaction time, hypotonia, coma Bulging fontanelle Respiratory rate > 60/min Severe chest indrawing Apnea ( > 15s) or bradypnea (respiratory rate < 20/min) Hypothermia ( < 35.5ºC) or fever (axillary temperature > 37.5ºC) Periumbilical erythema Crepitations/crackles on pulmonary auscultation	Purulent eye discharge Diarrhea/vomiting Hepatosplenomegaly Jaundice Numerous skin pustules Difficulty or refusal to suckle Acute crying/irritability
**Criteria for inclusion: 1 category A sign OR 2 category B signs**	

### Study procedures

After obtaining written informed consent, a trained study research nurse completed a standardized questionnaire that recorded infant and maternal demographics, maternal pregnancy and delivery course, history of the infant’s illness, and antimicrobial use in both the mother and the infant. The study team member also obtained vital signs, weighed participants with a digital baby scale, and performed a focused physical examination on the infant. On the day of enrollment, venous blood was collected using sterile procedures, ethanol cleaning followed by iodine-based disinfection of the skin and inoculated into an aerobic blood culture bottle (BacT/ALERT PF Plus; bioMerieux, Marcy l’Etoile). The target blood volume drawn was based upon patient weight and the study team attempted to obtain a minimum of 2 mL for culture [17]. A study team member recorded antimicrobials prescribed during the hospitalization and vital status on days 2, 7, and at 28 post-enrollment for those still admitted in the hospital.

### Laboratory methods

Study samples were analyzed at the Kilimanjaro Clinical Research Institute Biotechnology Laboratory. The volume of blood submitted for culture was determined by comparing the weight of blood culture bottles pre- and post-collection. Blood cultures were analyzed using the BacTAlert 3D Microbial Detection system (bioMérieux, Marcy l’Etoile, France) with a maximum incubation period of five days. Organisms were identified by Gram-stain, growth on sub-culture solid media agar (blood, chocolate and MacConkey agar plates), and biochemical workup. Gram-positive organisms were identified through phenotypic methods including: observation of colony morphology, motility test, hemolysis zone, bacitracin susceptibility, optochin test, catalasereaction, coagulase reaction, and PYR reaction, following standardized procedure flow charts. For Gram-negative organisms, oxidase reaction and API 20E (bioMérieux, Marcy l’Etoile, France) and API NE (bioMérieux, Marcy l’Etoile, France) were used for organism identification. Yeast were identified by Gram-stain and not speciated further. Antimicrobial susceptibility testing was manually performed using disk diffusion methods according to the Clinical Laboratory Standards Institute (CLSI; Wayne, Pennsylvania, USA) [18]. Susceptibility interpretations were based on the 2021 CLSI guidelines and interpretive criteria, classified as susceptible, intermediate, or resistant [18]. Extended spectrum beta-lactamase (ESBL) production by Gram-negative isolates was tested using the disk diffusion clavulanate inhibition test [18]. Blood culture results were shared with clinical teams and added to patient charts.

### Definitions and study outcomes

Our study included neonates, defined as infants 0–28 days of life, and very young infants, 29–60 days of life. Herein we will refer to the syndrome as neonatal sepsis and to all enrolled participants as neonates as this was a study conducted in the neonatal ward and we predominantly enrolled infants ≤  28 days old. Gestational ages were categorized using Ballard scoring: term infants were ≥  38 weeks, while preterm infants were <  38 weeks. Preterm infants were further classified as moderate-late preterm, 32-37 weeks; very preterm, 28–32 weeks; and extremely preterm, <  28 weeks. BSI was defined as blood culture isolation of established neonatal pathogens, including yeast and coagulase-negative *Staphylococcus* spp. (CoNS) [9,19–21]. The following were considered contaminants: *Micrococcus* sp., *Corynebacteria* sp. (other than *C. diphtheriae* or *C. jeikeium*), *Bacillus* sp. (other than *B. anthracis*), *Lactobacillus* sp., and viridans group *Streptococcus* sp. EO-BSI was defined as BSI occurring day of life (DOL) 0 through DOL 2, where DOL 0 is the day of birth. LO-BSI was defined as BSI occurring on or after DOL 3. Hospital-onset BSI (HO-BSI) was defined as a positive blood culture for bacteria or yeast, including CoNS, on day 4 or later of hospitalization at KCMC [22]. Antibacterials prescribed were grouped using the WHO Access, Watch, and Reserve (AWaRe) classification of antibacterials [23]. Briefly, antibacterials are divided into three categories—Access, Watch, and Reserve—based on clinical necessity and resistance profile [23]. Days of therapy refer to the number of days that a participant was prescribed an antibacterial, regardless of the dose or frequency of administration.

Effectiveness of prescribed antibacterials was determined through manual data review. Effective therapy was defined as prescription of an antibacterial with demonstrated activity based upon phenotypic susceptibility testing of the isolate or based upon CLSI guidance that susceptibility can be assumed. Ineffective therapy was defined as one or more of the following: (1) participant was prescribed an antibacterial without adequate activity against the microbiologically identified organism (e.g., ceftriaxone for *Pseudomonas* spp.) or intrinsic resistance (e.g., vancomycin for a gram-negative bacteria), (2) no antimicrobial was prescribed to the participant, (3) the microbiologically identified organism generally has susceptibility to the prescribed antibacterial but the specific isolate demonstrated phenotypic resistance against the antibacterial the participant was prescribed (e.g., receiving ceftriaxone and *E. coli* isolate susceptibility testing demonstrated resistance to third generation cephalosporins) or (4) the participant was prescribed effective antibacterial therapy for fewer than 7 days, a recommended treatment duration by WHO for neonatal BSI [10].

### Statistical analyses

Statistical analyses were performed with R software, version 4.4.1. The study population used for all analyses was enrolled infants with blood culture data. Descriptive statistics were performed with continuous variables expressed as median and interquartile range (IQR) or mean and 95% confidence interval (CI). Categorical variables are expressed as frequencies. Differences between infants with BSI and those without BSI were assessed using Pearson’s Chi-square test for categorical variables and the Wilcoxon Rank Sum Test for continuous variables. The association between variables of interest and odds of BSI was explored using univariable and multivariable logistic regression. Co-linearity was evaluated using the Variance Inflation Factor. Statistical significance was evaluated at p-values of < 0.05.

### Research ethics

This study was approved by the Kilimanjaro Christian Medical University College of Tumaini University Health Research Ethics Committee (Certificate number: 2577), the Tanzania National Institute for Medical Research National Health Research Ethics Coordinating Committee (NIMR/HQ/R.8a/Vol. IX/4085), and an Institutional Review Board of the Duke University Health System (Pro00109919). Written informed consent was obtained from the participant’s parent or guardian. Additional information regarding the ethical, cultural, and scientific considerations specific to inclusivity in global research is included in the Supporting Information (S1 File).

## Results

### Clinical and demographic characteristics of study participants

**Fig 1:**
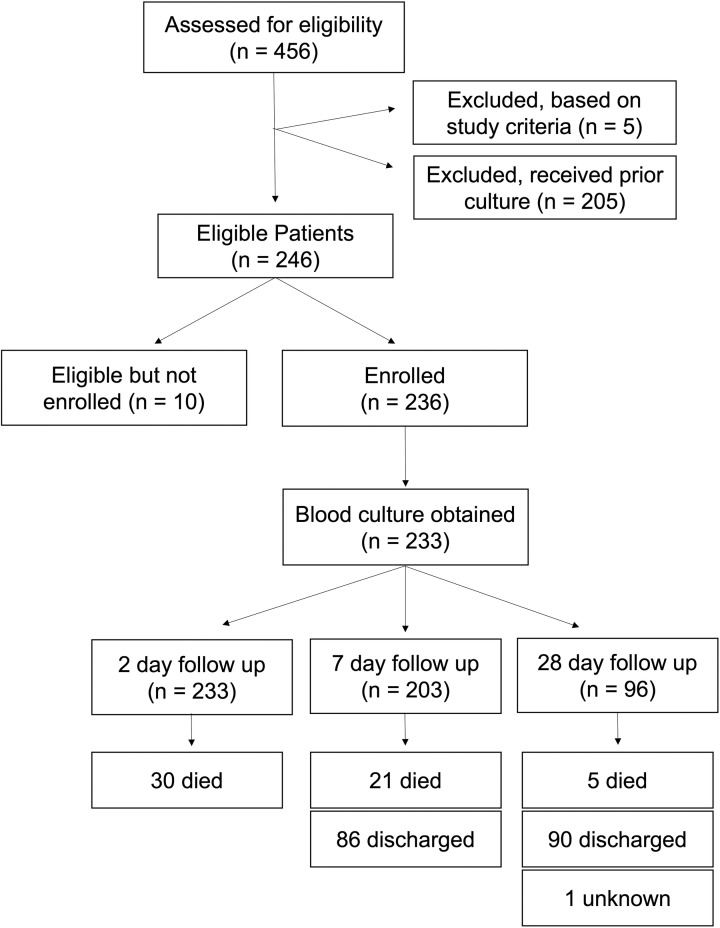
Flow chart showing the steps in the screening, enrollment, and data collection from the study population of neonates and infants with suspected sepsis in the neonatal ward of Kilimanjaro Christian Medical Centre from September 2022 through April 2023.

Among the 456 infants who were screened during the study period, 246 (53.9%) met eligibility criteria (**Fig 1**). Of those eligible, 236 (95.9%) infants were enrolled, and blood cultures were obtained from 233 (98.7%) participants by the study team. Among the 233 blood cultures, the median collected volume was 3.0 mL (IQR: 0) and 229 (98.3%) of collected cultures had volumes ≥  2mL. There were 112 positive cultures, of which 6 (5.4%) had organisms that were determined to be contaminants. Participants’ demographic and clinical characteristics are detailed in **Table 2**. Of the 233 participants with a blood culture 96 (41.2%) were female, and participants were a mean of 2 (IQR: 1, 6) days of age at the time of enrollment. There were 105 (45.1%) participants born at term, 81 (34.8%) moderate-late preterm, 35 (15.0%) very preterm, and 12 (5.2%) extremely preterm. Of those, 130 (55.8%) had birthweight of <  2,500 grams, and 143 (61.4%) required respiratory support at the time of enrollment. Among those participants, 112 (48.1%) of 233 were born at another hospital, 101 (43.3%) at KCMC, and 20 (8.6%) at home. Of births, 152 (65.2%) of 233 occurred via vaginal delivery, and 61 (26.2%) mothers reported prolonged rupture of membranes ≥  18 hours. Signs and symptoms of sepsis noted among study participants at the time of enrollment are described in S1 Table.

**Table 2:** Clinical and demographic characteristics of study participants, Kilimanjaro Christian Medical Centre, Tanzania, 2022–23.

Characteristics	All infants with blood culture (N = 233)		Infants without BSI (N = 127)		Infants with early-onset BSI (N = 50)		Infants with late-onset BSI (N = 56)	
Female sex, n (%)	96	(41.2)	54	(42.5)	19	(38.0)	23	(41.1)
Infant age at enrollment, median (IQR), days	2	(1–6)	2	(1–6)	1	(1–2)	6	(3–13)
Infant age category, n (%)								
Neonate, 0–28 days	228	(97.9)	127	(100.0)	50	(100.0)	51	(91.1)
Newborn, 29–60 days	5	(2.1)	0	(0.0)	0	(0.0)	5	(8.9)
Gestational age, n (%)								
Term, ≥ 38 weeks	105	(45.1)	58	(45.7)	19	(38.0)	28	(50.0)
Moderate–late preterm, 32–37 weeks	81	(34.8)	46	(36.2)	17	(34.0)	18	(32.1)
Very preterm, 28–32 weeks	35	(15.0)	17	(13.2)	12	(24.0)	6	(10.7)
Extremely preterm, < 28 weeks	12	(5.2)	6	(4.7)	2	(4.0)	4	(7.1)
Birthweight < 2500 grams, n (%)	130	(55.8)	69	(54.3)	31	(62.0)	28	(50.0)
Birthplace, n (%)								
KCMC	101	(43.3)	62	(48.8)	22	(44.0)	17	(30.4)
Home	20	(8.5)	9	(7.1)	4	(8.0)	7	(12.5)
Other hospital	112	(48.1)	56	(44.1)	24	(48.0)	32	(57.1)
Vaginal delivery, n (%)	152	(65.2)	86	(67.7)	31	(62.0)	35	(62.5)
Maternal age, median (IQR), years	27	(22–32)	26	(22–32)	30	(25–34)	27	(22–31)
Number of prior pregnancies, n (%)^1^								
0	84	(35.7)	54	(42.5)	9	(18.4)	18	(32.1)
1	66	(28.0)	32	(25.2)	12	(24.4)	22	(39.3)
2	35	(14.9)	20	(15.7)	6	(12.2)	9	(16.1)
3 +	50	(21.3)	21	(16.5)	22	(44.9)	7	(12.5)
Number of doses of maternal malaria prophylaxis received, n (%)^2^								
0	31	(13.3)	15	(11.9)	10	(20.0)	6	(10.7)
1–2	99	(42.6)	54	(42.9)	21	(42.0)	24	
3–4	102	(43.9)	57	(45.2)	19	(38.0)	26	(46.4)
Maternal HIV infection, n (%)	6	(2.5)	3	(2.4)	1	(2.0)	2	(3.6)
Maternal antibacterials during pregnancy, n (%)	59	(25.3)	30	(23.6)	13	(26.0)	16	(28.6)
Maternal antibacterials during delivery, n (%)	101	(42.9)	54	(42.5)	22	(44.0)	24	(42.9)
Prolonged rupture of membranes ≥ 18 hours, n (%)^3^	61	(26.5)	39	(31.2)	13	(26.0)	9	(16.4)
Respiratory support at enrollment, n (%)	143	(61.4)	69	(54.3)	40	(80.0)	34	(60.7)
Receipt of antibacterials prior to blood culture, n (%)	221	(94.8)	121	(95.3)	45	(90.0)	55	(98.2)
Receipt of antibacterials during admission, n (%)	231	(99.1)	126	(99.2)	49	(98.0)	56	(100.0)
Number of antibacterials prescribed, median (IQR)	2	(2–3)	2	(2–3)	2	(2–4)	2	(2–4)
Days of therapy, median (IQR)	7	(5–9)	7	(5–9)	7	(5–9)	7	(5–9)
Length of stay, median (IQR), days	7	(4,13)	7	(4,10)	6	(4,10)	8	(5,19)
Died while hospitalized, n (%)	56	(24.0)	27	(21.3)	11	(22.0)	18	(32.1)

Abbreviations: Kilimanjaro Christian Medical Centre (KCMC), bloodstream infection (BSI), day of life (DOL), interquartile range (IQR).

^1^Prior pregnancy data was missing from one infant with early-onset BSI.

^2^Doses of maternal malaria prophylaxis received was missing from one infant without BSI.

^3^Prolonged rupture of membranes >  18 hours data were missing from two infants without BSI and one infant with late-onset BSI.

### BSI prevalence and antimicrobial resistance patterns of organisms associated with BSI

Among the 233 infants with blood cultures, 106 (45.5%) had BSI (**Table 3**). Gram-negative bacteria (GNB) were identified in 40 infants, Gram-positive bacteria in 58 infants, and yeast in 8 infants. All 40 Gram-negative isolates had ESBL production and universal resistance to ampicillin and ceftriaxone, 12 (30.0%) exhibited phenotypic gentamicin resistance, and 1 (2.5%) exhibited phenotypic carbapenem resistance. Among the 58 Gram-positive bacterial isolates, 13 (22.4%) exhibited phenotypic resistance to oxacillin. Detailed antibacterial susceptibility data are shown in S2 Table. Median time to blood culture positivity was 11.8 (IQR: 10.7, 15.9) hours for GNB, 18.2 (IQR: 15.7, 27.0) hours for CoNS, 14.9 (IQR: 14.6, 15.4) hours for *S. aureus*, and 73.4 (IQR: 69.9, 81.2) hours for yeast*.*

**Table 3:** Frequency and antimicrobial resistance patterns of organisms associated with early-onset and late-onset bloodstream infections (BSI) among study participants, Kilimanjaro Christian Medical Centre, Tanzania, Tanzania, 2022-23.

Organism		Early-onset BSI N = 50, n (%)	Early-onset BSI with ESBL^†^, n (%)	Late-onset BSI N = 56, n (%)	Late-onset BSI with ESBL^†^, n (%)	Total BSI N = 106, n (%)	Total with ESBL^†^, n (%)
Gram-negative bacteria		21 (42.0)	21 (100.0)	19 (33.9)	19 (100.0)	40 (37.7)	40 (100.0)
*Klebsiella* spp.		21 (42.0)		15 (26.8)		36 (33.9)	
* K. pneumoniae*		21 (42.0)		14 (25.0)		35 (33.0)	
* K. oxytoca*		0 (0.0)		1 (1.8)		1 (0.9)	
*Acinetobacter baumannii*		0 (0.0)		2 (3.6)		2 (1.9)	
*Escherichia coli*		0 (0.0)		2 (3.6)		2 (1.9)	
**Organism**		**Early-onset BSI** **N = 50, n (%)**	**Early-onset BSI with oxacillin resistance** ^†^ **, n (%)**	**Late-onset BSI** **N = 56, n (%)**	**Late-onset BSI with oxacillin resistance, n (%)**	**Total BSI** **N = 106, n (%)**	**Total with oxacillin resistance**^†^, **n (%)**
Gram-positive bacteria		24 (48.0)	3 (12.5)	34 (60.7)	10 (29.4)	58 (54.7)	13 (22.4)
	*S. aureus*	0 (0.0)	0 (0.0)	3 (5.4)	1 (33.3)	3 (2.8)	1 (33.3)
	Coagulase-negative Staphylococcus^*^	24 (48.0)	3 (12.5)	31 (55.4)	9 (29.0)	55 (51.9)	12 (21.8)
**Organism**		**Early-onset BSI** **n = 50, n (%)**	**Early-onset BSI with resistance**	**Late-onset BSI** **n = 56, n (%)**	**Late-onset BSI with resistance**	**Total BSI** **n = 106, n (%)**	**Total with resistance**
Yeast^*^		5 (10.3)	–	3 (5.4)	–	8 (7.5)	–

Abbreviations: bloodstream infection (BSI); extended-spectrum beta lactamase (ESBL). All dashes mean not applicable. There are no missing data.

* Speciation by biochemical phenotypic methods is pending for coagulase-negative *Staphylococcus* and yeast isolates.

† Proportions for ESBL and oxacillin-resistant BSIs were calculated using the number of early-onset or late-onset BSIs for each organism as the denominator.

**Fig 2:**
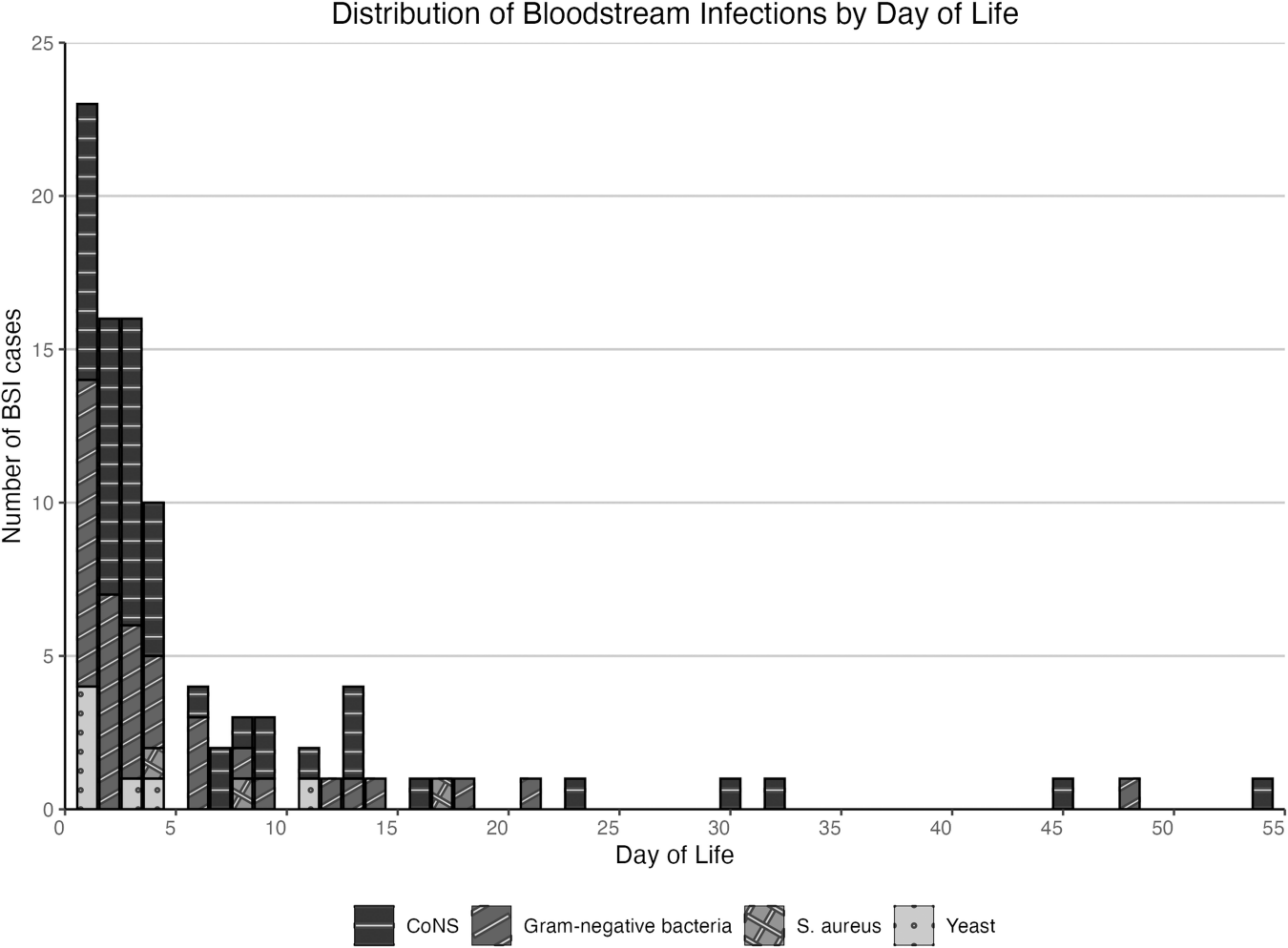
Bloodstream infections (BSI) by day of life (DOL) on which the blood culture was obtained. Day of birth is DOL 0. Early-onset BSI cases (n =  50) occurred on DOL 0-2, late-onset BSI (n = 56) occurred on DOL 3 or later. Abbreviations: CoNS =  coagulase-negative Staphylococcus.

BSIs occurred from DOL 0 through DOL 54. Of 106 BSIs, 82 (77.4%), occurred within the first week of life (**Fig 2**). EO-BSI occurred in 50 infants with 21 (42.0%) due to ESBL-producing *Klebsiella* spp., 24 (48.0%) to CoNS, and 5 (10.0%) to yeast. LO-BSI occurred in 56 infants, with 19 (33.9%) of those due to ESBL-producing GNB bacteria, 31 (55.4%) to CoNS, 3 (5.4%) to *S. aureus*, and 3 (5.4%) to yeast. Among the 50 infants with EO-BSI, 36 (72.0%) were term or moderate-late preterm, and 14 (28.0%) were very or extremely preterm. Of the 56 LO-BSIs, 46 (82.1%) were in term or moderate-late preterm infants and 10 (17.9%) were in very or extremely preterm infants. Of 50 infants with EO-BSI, 22 (44.0%) were born at KCMC, 4 (8.0%) were born at home, and 24 (48.0%) were born at other hospitals and transferred to KCMC. Among infants with LO-BSI, 17 (30.4%) of 56 were born at KCMC, 7 (12.5%) were born at home, and 32 (57.1%) were born at other hospitals and transferred to KCMC.

**Fig 3:**
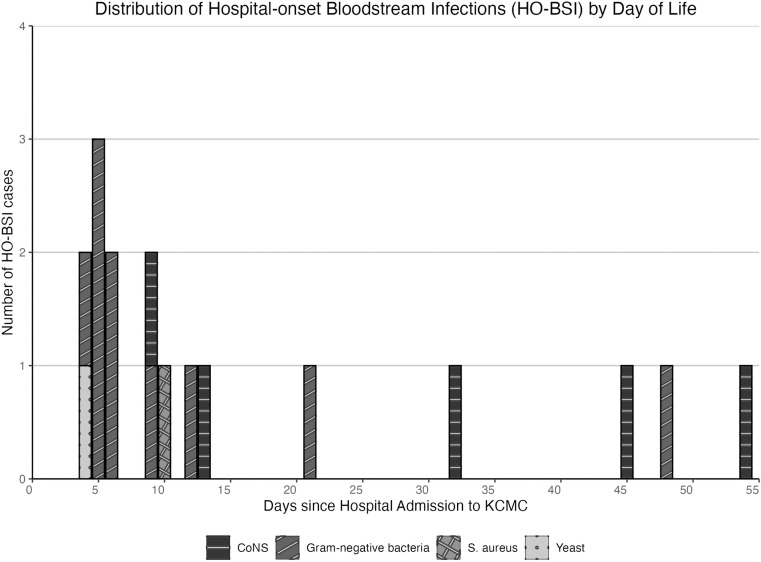
Hospital onset-bloodstream infections (HO-BSI) by days since admission to Kilimanjaro Christian Medical Centre (KCMC). HO-BSI (n = 17) was defined as bloodstream infection occurring on day 4 or later of hospitalization at KCMC. Abbreviations: CoNS =  coagulase-negative *Staphylococcus*.

HO-BSI was identified in 17 infants, occurring from 4 days through 54 days of admission to KCMC (**Fig 3**). HO-BSI was more common in infants with birthweight <  2,500 grams compared to those with non-HO-BSI (p =  0.012) (S3 Table). Of infants with HO-BSI, 4 (23.5%) of 17 were born at KCMC, 3 (17.6%) were born at home, and 10 (58.8%) were born at other hospitals and transferred to KCMC. The occurrence of HO-BSI was highest in the first 2 weeks of admission to KCMC with 10 (58.8%) of 17 BSIs occurring within that period. Among those with HO-BSI, 10 (58.8%) were due to GNB with 9 (52.9%) due to *Klebsiella* spp., and 1 (5.9%) due to *E. coli*. Of the remaining HO-BSI, 5 (29.4%) were due to CoNS, 1 (5.9%) to *S. aureus*, and 1 (5.9%) to yeast.

### Antibacterials prescribed to participants during admission at KCMC

During admission, 231 (99.1%) of 233 participants received antibacterials, with 221 (94.8%) receiving them before blood culture. The most prescribed antibacterials were ampicillin-cloxacillin (177, 76.6%) and gentamicin (174, 75.3%). Access antibacterials including gentamicin, metronidazole, or penicillin were prescribed to 188 (81.4%) of 231 infants on antibacterials. Watch antibacterials including, ceftriaxone, ciprofloxacin, meropenem, piperacillin-tazobactam or vancomycin, were prescribed to 125 (54.1%) of 231 infants. Meropenem was prescribed to 88 (38.1%) and vancomycin to 65 (28.1%) of 231 infants. No reserve antibacterials were prescribed. The median duration of prescribed therapy was 7 days (IQR: 5,9) overall and participants were prescribed a median of 2 (IQR: 2,3) antibacterials during the admission.

Among the 106 participants with BSI, 50 (47.2%) were prescribed ineffective antibacterial therapy as detailed in S4 Table. Of these 50 infants, 8 (16.0%) died and 42 (84.0%) were discharged or remained alive at day 28 follow up. Among those who were prescribed ineffective therapy, 19 (38.0%) of 50 were prescribed effective antibacterials for fewer than 7 days, 15 (30.0%) had BSI due to an organism that generally has susceptibility to the prescribed antibacterial, but the specific isolate demonstrated resistance to the antibacterial prescribed, 15 (30.0%) were prescribed an antibacterial without activity against the identified organism, and 1 was not prescribed antibacterials. There were 4 deaths among infants who were prescribed an antibacterial that was ineffective because of the organism’s resistance profile, 3 deaths among infants who were prescribed antibacterials without adequate activity against the identified organism, and 1 death of an infant who did not receive 7 days of effective therapy.

### Length of stay and mortality outcomes

Length of stay for all participants and those with BSI are detailed in **Table 2**, and for infants with HO-BSI in S3 Table. The all-cause mortality among infants with blood culture data was 56 (24.0%) of 233. Among the 56 infants who died, 29 (51.8%) had BSI; 11 (19.6%) infants with EO-BSI, and 18 (32.1%) with LO-BSI. Of the 17 infants with HO-BSI, 7 (41.2%) died while hospitalized. The mortality among those with BSI was 29 (27.4%) of 106, compared to 27 (21.3%) of 127 among those without BSI (p =  0.35). The mortality among those with EO-BSI was 11 (22.0%) of 50, and among those with LO-BSI was 18 (32.1%) of 56 (p =  0.24). The mortality among those with GNB BSI was 15 (37.5%) of 40, and among those with CoNS BSI was 12 (21.8%) of 55 (p =  0.94). Among the 8 infants who died and were prescribed ineffective therapy, 4 (50.0%) had GNB BSI. Of these 4 infants, 3 (75.0%) were prescribed an antibacterial that was ineffective because of the GNB’s resistance profile. One of the three aforementioned deaths occurred in an infant infected with a carbapenem-resistant GNB (*Acinetobacter baumannii*

### Univariable and multivariable logistic regression

#### Odds of BSI, early-onset and late-onset BSI.

The results of univariable and multivariable risk factor analyses for BSI, EO-BSI, and LO-BSI compared to infants without BSI are given in **Table 4**. Preterm gestation and maternal antibacterials during pregnancy were excluded from the multivariable models for EO-BSI and LO-BSI due to co-linearity with other variables. Multivariable analysis showed infants needing respiratory support at admission had increased odds of BSI (aOR 1.89, 95% CI 1.05 – 3.44) and EO-BSI (aOR 3.12, 95% CI 1.44 – 7.28). Infants born at KCMC had lower odds of LO-BSI compared to those born at another hospital or at home (aOR 0.38, 95% CI 0.17 – 0.84).

**Table 4:** Associations between infant and maternal characteristics and early-onset and late-onset bloodstream infections (BSI) among study participants, Kilimanjaro Christian Medical Centre, Tanzania, Tanzania, 2022-23.

Characteristic	BSI of any type (n = 106)						Early-onset BSI (n = 50)						Late-onset BSI (n = 56)					
	OR	95% CI	p-value	aOR	95% CI	p-value	OR	95% CI	p-value	aOR	95% CI	p-value	OR	95% CI	p-value	aOR	95% CI	p-value
Preterm, < 38 weeks gestational age	1.06	0.63–1.77	0.84	0.92	0.33–2.51	0.87	1.37	0.71–2.71	0.355	–	–	–	0.84	0.45–1.58	0.59	–	–	–
Birthweight < 2,500 grams	1.14	0.68–1.92	0.62	1.25	0.46–3.45	0.67	1.37	0.71–2.71	0.355	1.14	0.54–2.43	0.73	0.97	0.52–1.83	0.92	1.28	0.63 – 2.62	0.49
Born at KCMC	0.61	0.36–1.03	0.07	0.52	0.27–1.01	0.05	0.82	0.42–1.59	0.56	0.79	0.34–1.84	0.59	**0.46**	**0.23–0.88**	**0.021**	**0.38**	**0.17 –** **0.84**	**0.02**
Vaginal delivery	0.79	0.46–1.35	0.39	0.74	0.30–1.81	0.52	0.78	0.40–1.55	0.47	0.77	0.24–2.39	0.65	0.79	0.41–1.54	0.49	0.75	0.24 – 2.29	0.62
Prolonged rupture of membranes ≥ 18 hours	0.58	0.32–1.06	0.08	0.67	0.34–1.28	0.23	0.77	0.36–1.59	0.50	0.89	0.39–1.98	0.79	**0.43**	**0.18–0.93**	**0.04**	0.54	0.22 – 1.24	0.16
Maternal antibacterials during pregnancy	1.22	0.67–2.20	0.51	1.54	0.81–2.95	0.19	1.14	0.52–2.38	0.74	–	–	–	1.29	0.63–2.61	0.48	–	–	–
Maternal antibacterials during delivery	1.04	0.61–1.75	0.89	1.13	0.45–2.79	0.79	1.06	0.55–2.05	0.86	0.97	0.29–3.15	0.96	1.01	0.53–1.91	0.97	1.2	0.38 – 3.68	0.75
Respiratory support on admission	**1.94**	**1.14–3.37**	**0.02**	**1.89**	**1.05–3.44**	**0.04**	**3.36**	**1.60–7.64**	**0.002**	**3.12**	**1.44–7.28**	**0.006**	1.30	0.69–2.49	0.42	1.13	0.57 – 2.26	0.73

Abbreviations: bloodstream infection (BSI), odds ratio (OR), 95% confidence interval (CI), adjusted odds ratio (aOR), Kilimanjaro Christian Medical Centre (KCMC)

#### Odds of CoNS BSI and GNB BSI.

The results of univariable and multivariable risk factor analyses for CoNS and GNB BSI compared to infants without BSI are given in **Table 5**. The multivariable model for CoNS BSI identified decreased odds of infection among infants born at KCMC compared to those who were born at an outside hospital or at home (OR 0.43 95% CI 0.19 – 0.96). The multivariable model for GNB BSI identified increased odds of infection among infants who required respiratory support upon admission compared to those who did not (OR 5.35, 95% CI 1.97 – 17.51).

**Table 5:** Associations between infant and maternal characteristics and bloodstream infection (BSI) by organism among study participants, KCMC, Tanzania, 2022-23.

Characteristic	BSI with CoNS (n = 55)						BSI with GNB (n = 40)					
	OR	95% CI	p-value	aOR	95% CI	p-value	OR	95% CI	p-value	aOR	95% CI	p-value
Preterm, < 38 weeks gestational age	0.56	0.29–1.06	0.08	0.52	0.17–1.60	0.26	**2.52**	**1.17–5.83**	**0.023**	3.31	0.68 –18.80	0.15
Birthweight < 2,500 grams	0.70	0.37–1.32	0.27	1.47	0.48–4.68	0.5	**2.22**	**1.04–4.99**	**0.045**	0.76	0.14–3.72	0.74
Born at KCMC	**0.43**	**0.21–0.84**	**0.02**	**0.43**	**0.19–0.96**	**0.04**	0.95	0.46–1.93	0.88	0.54	0.19–1.46	0.24
Vaginal delivery	0.90	0.47–1.78	0.77	0.8	0.25–2.48	0.7	**0.48**	**0.23–0.98**	**0.045**	0.42	0.10–1.58	0.21
Prolonged rupture of membranes ≥ 18 hours	0.44	0.19–0.96	0.05	0.59	0.24–1.35	0.22	0.64	0.27–1.43	0.29	0.72	0.27–1.79	0.49
Maternal antibacterials during pregnancy	1.21	0.58–2.47	0.60	1.18	0.53–2.56	0.68	0.94	0.38–2.13	0.88	1.68	0.60–4.64	0.31
Maternal antibacterials during delivery	0.90	0.47–1.71	0.75	1.02	0.32–3.19	0.98	1.49	0.73–3.07	0.27	0.94	0.22–3.78	0.93
Respiratory support on admission	1.09	0.58–2.07	0.80	1.08	0.54–2.18	0.83	**5.88**	**2.34–18.01**	**0.001**	**5.35**	**1.97 –17.51**	**0.002**

Abbreviations: bloodstream infection (BSI), coagulase-negative *Staphylococcus* (CoNS), Gram-negative bacteria (GNB), odds ratio (OR), 95% confidence interval (CI), adjusted odds ratio (aOR), Kilimanjaro Christian Medical Centre (KCMC).

## Discussion

We demonstrate that *K. pneumoniae* and CoNS BSI were common in the neonatal ward of a tertiary referral hospital in northern Tanzania. The prevalence of resistance to WHO-recommended first- and second-line antibacterials was high among all GNB isolates. Nearly one-quarter of infants with suspected sepsis died, more than half of whom had BSI. Our findings add to growing evidence that neonatal BSI due to multi-drug resistant bacteria have a substantial impact on neonatal mortality in sub-Saharan Africa. Optimization of diagnosis, empiric treatment regimens, and infection prevention and control are urgently needed to improve outcomes.

GNB, predominately *K. pneumoniae,* were implicated in 40 (37.7%) of the 106 BSIs. This proportion is similar to or slightly higher than data from elsewhere in sub-Saharan Africa [6–8]. *Klebsiella* species were identified as the cause of 19% of neonatal BSIs in Eastern African studies published between 2008 and 2018 [8]. *E. coli* was implicated in only two BSIs in our study, whereas it has been previously identified as the cause of 10% of BSIs in Eastern Africa [8]. At KCMC, more than half of HO-BSIs were caused by GNB, raising concern for a potential nosocomial source within the neonatal ward. Among the *K. pneumoniae* isolates, resistance was common to WHO-recommended therapies for neonatal sepsis. One-third exhibited phenotypic resistance to gentamicin, the first-line recommended therapy with ampicillin, and all were ESBL-producing with universal phenotypic resistance to ceftriaxone, the second-line therapy recommended by WHO. These epidemiological and antimicrobial resistance patterns align with others from Tanzania [24,25]. There was one carbapenem-resistant GNB isolated in our cohort and hyper-resistant GNB are emerging in LMICs [26].

CoNS was the most commonly isolated Gram-positive organism and was found in 55.4% of LO-BSIs. In many clinical contexts these organisms are considered contaminants, as they commonly reside on skin; but among neonates, CoNS are established causes of infections, particularly those born at very low birth weight (less than 1,500g) and are implicated clonal outbreaks in neonatal wards [27–30]. *S. aureus* was rare, accounting for <  3% of BSI in this study;;and there were no isolations of GBS. Our findings differ from other studies in Eastern Africa that identified *S. aureus* in 20% of BSIs. Approximately 80% of Gram-positive isolates were susceptible to oxacillin. In high-income settings, GBS is a common cause of EO-BSI and is treated with ampicillin, yet, multiple single-center studies from African LMICs have described low GBS prevalence ranging from 0 to 6% [8,24,31–33]. However, there may be substantial regional variability of maternal GBS colonization across Africa, and GBS may be an underrecognized cause of neonatal infection and stillbirth in some regions [34,35]. Based on the local epidemiology suggested by this study, it may be prudent for clinicians in northern Tanzania to consider alternative agents, such as an anti-staphylococcal penicillin, cefazolin, or vancomycin for Gram-positive coverage based on drug availability, patient risk factors, and the infant’s clinical status.

Most infants enrolled in our study were prescribed ampicillin-cloxacillin and gentamicin during their admission. The WHO discourages use of ampicillin-cloxacillin as there is little clinical benefit of fixed combinations over using the individual agents and these antibacterials may contribute to development of AMR [23]. Access group antibacterials were the most frequently prescribed, but more than half of infants were prescribed a Watch group antibacterial, including meropenem and vancomycin prescribed for 38.1% and 28.1%, respectively. These prescribing patterns are likely influenced by the need to escalate treatment due to patients’ clinical deterioration and by reduced clinical response to first- and second-line therapies, rather than by local resistance data [6,9]. Globally, the current WHO-recommended therapies are increasingly ineffective against common neonatal BSI pathogens and in our cohort, 30% of infants with BSI were prescribed an antibacterial that was ineffective because of the organism’s resistance profile [6,36]. Alternative empiric regimens for neonatal sepsis should be considered based on regional and local epidemiology, while also taking antimicrobial stewardship into account to minimize the risk of future highly resistant infections. Rapid laboratory diagnostics and context-specific risk stratification tools are necessary to quickly and accurately identify neonatal BSI and judiciously escalate and de-escalate use of broad-spectrum empiric therapy in infants [5].

The organisms implicated in EO-BSI, LO-BSI, and HO-BSI were similar in our study. This similarity could suggest that the maternal flora and community microbiome may resemble nosocomial pathogens in the hospital environment. Microbial genomic data would be valuable in addressing these epidemiologic questions and guiding future interventions at KCMC. There is an urgent need for effective strategies to prevent early-onset and late-onset infections by addressing maternal intrapartum disease through prevention and early treatment and by improving infection prevention and control measures within neonatal wards [37]. For example, maternal capsular polysaccharide and O lipopolysaccharide vaccines to prevent neonatal *K. pneumoniae* infection via placental antibody transfer are in development [38]. These vaccines could have the largest impact in African countries and could markedly reduce overall burden of sepsis, antibacterial use, and nosocomial outbreaks [39]. Infection prevention and control bundles that include hand-hygiene compliance, environmental and equipment cleaning, and device management have successfully reduced sepsis and death in low-resource settings [40,41].

Nearly one-quarter of all infants enrolled in the study died, and of those, half had BSI. Among the 8 infants who died and also were prescribed ineffective therapy, half had a GNB; and of those with GNB, three-quarters were prescribed an antibacterial that was ineffective due to the bacteria’s resistance profile. There was no statistically significant difference in mortality between EO-BSI and LO-BSI, or between GNB BSI and CoNS BSI. Infants who required respiratory support at admission had increased odds of BSI, specifically GNB BSI, compared to those who did not require respiratory support. It is possible that infants who required respiratory support, or those born at a tertiary referral hospital, had other complications of prematurity or low birth weight that contributed to mortality. However, the majority of LO-BSI and HO-BSI occurred in term or moderate-late preterm infants who survived the highest risk neonatal period and are typically at lower risk of necrotizing enterocolitis and respiratory distress syndrome [42]. LO-BSI could also have occurred due to nosocomial infection with use of medical devices such as nasal cannulas.

We acknowledge limitations to our study. The sample size was small, and the findings are representative of a single center. However, these data are relevant to inform practice at KCMC and potentially other Tanzanian referral hospitals. The data may not be fully representative of the admitted patient population as our inclusion criteria and study procedures meant that 205 overnight and weekend admissions were excluded from our study. Nearly all infants received antibacterials before blood culture was drawn, which may have limited the diagnostic yield of blood culture. There were several reasons why obtaining a blood culture before starting antibacterials was not always feasible. First, collecting blood cultures in infants can be technically challenging and may require multiple attempts. To avoid delays in care, antibacterials are often administered upon admission to the ward. Second, there were clinical scenarios where the infant received antibacterials prior to study enrollment. For instance, infants born outside of KCMC may have already been given antibacterials before transfer. In some cases, empiric antibacterials might have been administered overnight without obtaining a blood culture, and the infant could have been enrolled in the study the following day. Additionally, an infant might have had a blood culture taken and received antibacterials outside of the study’s enrollment hours, only to later develop signs of sepsis, which then prompted enrollment in the study. Finally, while we educated clinicians to wait until after obtaining a blood culture to start antibacterials when clinically safe, the study was observational and descriptive in nature. Thus, the research team did not directly influence clinical decisions. It is also possible that historical practices, such as the routine initiation of antibacterials upon admission, were followed, as this is standard practice for some clinicians in neonatal wards. While we classified all blood cultures with growth of CoNS as true BSI, we acknowledge that some of these isolations could represent skin contaminants rather than BSI. Repeat blood cultures, which can confirm BSI if positive, were not obtained. Nevertheless, as the median time to positivity for CoNS growth was less than 24 hours and CoNS is an established neonatal pathogen, our classification was an appropriate approach [4,20,43]. We were unable to work up yeast organisms further, limiting our description of these BSIs. Finally, we only report in-hospital mortality as we did not follow patients after discharge. To fully understand neonatal mortality in this setting, data on post-discharge outcomes would be useful.

Neonatal BSIs due to ESBL-producing *K.* pneumoniae and CoNS were highly prevalent in a tertiary referral hospital in northern Tanzania. Our findings underscore the need to mitigate preventable deaths from neonatal BSI through locally-contextualized, multifaceted interventions developed using quality improvement and antimicrobial stewardship frameworks. These interventions should include empiric therapy for infants guided by local epidemiology and resistance patterns, improving recognition and treatment of intrapartum infections in mothers, and robust infection prevention and control measures for neonatal wards.

## Supporting information

S1 Table Sepsis signs and symptoms noted among study participants at the time of study enrollment, Kilimanjaro Christian Medical Centre, Tanzania, 2022–23. (DOCX)

S2 Table Antibacterial susceptibility of bloodstream isolates obtained from enrolled participants, Kilimanjaro Christian Medical Centre, Tanzania, 2022–23. (DOCX)

S3 Table Characteristics of study participants with hospital-onset BSI, Kilimanjaro Christian Medical Centre, Tanzania, 2022–23. (DOCX)

S4 Table Adjudication of ineffective antibacterial therapy among participants with bloodstream infection, Kilimanjaro Christian Medical Centre, Tanzania, 2022–23. (DOCX)

S1 File Inclusivity in Global Research Questionnaire. (DOCX)
