# Supplementary material for: Clinical characteristics, antimicrobial resistance, and mortality of neonatal bloodstream infections in Northern Tanzania, 2022–2023
Source: PLoS One. 2025 Mar 25;20(3):e0319816. doi: 10.1371/journal.pone.0319816 (PMC11936297; doi:10.1371/journal.pone.0319816)
Supplement: S1 Table — (DOCX) [file pone.0319816.s001.docx]

**.Supplementary Table 1: Sepsis signs and symptoms noted among study participants at the time of study enrollment, Kilimanjaro Christian Medical Centre, Tanzania, 2022-23**

| **Sign or symptom** | | **Overall  (N = 233)** | | **No BSI  (N = 127)** | | **Early-onset BSI (N=50)** | | | **Late-onset BSI (N=56)** | | |
| --- | --- | --- | --- | --- | --- | --- | --- | --- | --- | --- | --- |
| Respiratory support (%) | | 143 | (61.4) | 69 | (54.3) | 40 | (80.0) | | 34 | (60.7) | |
| Type of respiratory support (%) | | | | | | | | | | | |
|  | Nasal cannula | 77 | (33.0) | 37 | (29.1) | 24 | | (48.0) | 16 | | (28.6) |
|  | Continuous positive airway pressure (CPAP) | 62 | (26.6) | 29 | (22.8) | 16 | | (32.0) | 17 | | (30.4) |
|  | Missing | 4 | (1.7) | 3 | (2.4) | 0 | | (0.0) | 1 | | (1.8) |
| Delayed perfusion ≥ 3 seconds (%) | | 7 | (3.0) | 2 | (1.6) | 1 | | (2.0) | 4 | | (7.1) |
| Retractions (%) | | | | | | | | | | | |
|  | None | 129 | (55.4) | 77 | (60.6) | 20 | | (40.0) | 32 | | (57.1) |
|  | Mild to moderate | 91 | (39.1) | 46 | (36.2) | 26 | | (52.0) | 19 | | (33.9) |
|  | Severe | 13 | (5.6) | 4 | (3.1) | 4 | | (8.0) | 5 | | (8.9) |
| Skin or mucosal findings (%) | | | | | | | | | | | |
|  | Petechiae | 1 | (0.4) | 1 | (0.8) | 0 | | (0.0) | 0 | | (0.0) |
|  | Erythema or cellulitis | 1 | (0.4) | 0 | (0.0) | 0 | | (0.0) | 1 | | (1.8) |
|  | Sclerema | 10 | (4.3) | 3 | (2.4) | 1 | | (2.0) | 6 | | (10.7) |
|  | Other | 20 | (8.6) | 10 | (7.9) | 2 | | (4.0) | 8 | | (14.3) |
| Alertness (%) | | | | | | | | | | | |
|  | Normal | 97 | (41.6) | 61 | (48.0) | 22 | | (44.0) | 14 | | (25.0) |
|  | Irritable | 27 | (11.6) | 12 | (9.4) | 5 | | (10.0) | 10 | | (17.9) |
|  | Lethargic | 109 | (46.8) | 54 | (42.5) | 23 | | (46.0) | 32 | | (57.1) |
| Birth asphyxia (%) | | 48 | (20.6) | 22 | (17.3) | 17 | | (34.0) | 9 | | (16.1) |
| Difficulty or refusal to suckle (%) | | 97 | (41.6) | 52 | (40.9) | 19 | | (38.0) | 26 | | (46.4) |
| Drowsiness, slow reaction time, hypotonia, coma (%) | | 90 | (38.6) | 40 | (31.5) | 20 | | (40.0) | 30 | | (53.6) |
| Bulging fontanelle (%) | | 4 | (1.7) | 2 | (1.6) | 1 | | (2.0) | 1 | | (1.8) |
| Convulsions (%) | | 22 | (9.4) | 11 | (8.7) | 7 | | (14.0) | 4 | | (7.1) |
| Redness around the umbilical cord (%) | | 3 | (1.3) | 3 | (2.4) | 0 | | (0.0) | 0 | | (0.0) |
| Respiratory rate > 60 breaths/minute (%) | | 68 | (29.2) | 29 | (22.8) | 23 | | (46.0) | 16 | | (28.6) |
| Severe chest indrawing (%) | | 35 | (15.0) | 13 | (10.2) | 11 | | (22.0) | 9 | | (16.1) |
| Apnea (> 15 seconds) or bradypnea (respiratory rate < 20 breaths/minute) (%) | | 33 | (14.2) | 13 | (10.2) | 11 | | (22.0) | 9 | | (16.1) |
| Hypothermia (axillary temperature < 35.5°C) or fever (axillary temperature ≥ 37.5°C) (%) | | 104 | (44.6) | 49 | (38.6) | 18 | | (36.0) | 37 | | (66.1) |
| Purulent discharge from the eyes (%) | | 4 | (1.7) | 2 | (1.6) | 0 | | (0.0) | 2 | | (3.6) |
| Crepitations/crackles on pulmonary auscultation (%) | | 18 | (7.7) | 6 | (4.7) | 6 | | (12.0) | 6 | | (10.7) |
| Excessive crying or irritability (%) | | 33 | (14.2) | 17 | (13.4) | 5 | | (10.0) | 12 | | (21.4) |
| Numerous skin pustules (%) | | 11 | (4.7) | 4 | (3.1) | 2 | | (4.0) | 5 | | (8.9) |
| Diarrhea or vomiting (%) | | 3 | (1.3) | 0 | (0.0) | 0 | | (0.0) | 3 | | (5.4) |
