# Supplementary material for: Clinical characteristics, antimicrobial resistance, and mortality of neonatal bloodstream infections in Northern Tanzania, 2022–2023
Source: PLoS One. 2025 Mar 25;20(3):e0319816. doi: 10.1371/journal.pone.0319816 (PMC11936297; doi:10.1371/journal.pone.0319816)
Supplement: S2 Table — (DOCX) [file pone.0319816.s002.docx]

**Supplementary Table 2: Antibacterial susceptibility of bloodstream isolates obtained from enrolled participants, Kilimanjaro Christian Medical Centre, Tanzania, 2022-23**

| **Organism**  **(n isolates)** | **Percentage of isolates susceptible (%)** | | | | | | | | | | | | | | | | |
| --- | --- | --- | --- | --- | --- | --- | --- | --- | --- | --- | --- | --- | --- | --- | --- | --- | --- |
|  | **AMP** | **AMC** | **AZM** | **CFZ** | **FOX** | **CAZ** | **AXO** | **CHL** | **CIP** | **ERY** | **GEN** | **MER** | **NAL** | **PEN** | **PIP** | **SXT** | **VAN** |
| **Gram-negative organisms (n = 40)** | | | | | | | | | | | | | | | | | |
| *Klebsiella pneumoniae* (35) | 0 | 60 | -- | -- | -- | 3 | 0 | 79 | 20 | -- | 79 | 100 | 86 | -- | -- | 0 | -- |
| *Klebsiella oxytoca* (1)^*^ | 0 | 0 | -- | -- | -- | 0 | 0 | -- | 0 | -- | -- | 100 | 100 | -- | -- | 0 | -- |
| *Escherichia coli* (2)^*^ | 0 | 100 | -- | 0 | -- | 0 | 0 | 50 | 50 | -- | 50 | 100 | 50 | -- | -- | 50 | -- |
| *Acinetobacter baumanii* (2)^*^ | 0 | -- | -- | -- | -- | 0 | 0 | -- | 50 | -- | 0 | 50 | -- | -- | -- | 50 | -- |
| **Gram-positive organisms (n = 58)** | | | | | | | | | | | | | | | | | |
| Coagulase-negative *Staphylococcus* (55) | -- | -- | -- | -- | 78 | -- | -- | 32 | -- | 15 | -- | -- | -- | 4 | -- | 19 | 98 |
| *Staphylococcus aureus* (3)^*^ | -- | -- | -- | -- | 66 | -- | -- | 100 | -- | 0 | -- | -- | -- | -- | -- | 0 | 100 |
| Abbreviations: ampicillin (AMP), amoxicillin-clavulanate (AMC), azithromycin (AZM), cefazolin (CFZ), cefoxitin (FOX), ceftazidime (CAZ), ceftriaxone (AXO), chloramphenicol (CHL), ciprofloxacin (CIP), erythromycin (ERY), gentamicin (GEN), nalidixic acid (NAL), penicillin (PEN), piperacillin (PIP), trimethoprim-sulfamethoxazole (SXT), vancomycin (VAN). All dashes mean susceptibility testing was not done for that antibacterial.  * According to the Clinical & Laboratory Standards Institute guidelines, a minimum of 30 diagnostic isolates are required to include the organism in an antibiogram. In our data, many identified organisms fall below this threshold. This is a limitation to the clinical utility of this antibiogram. Additionally these data are reflective of the single institution and the ward where our study was conducted. Thus, the data may not be generalizable. | | | | | | | | | | | | | | | | | |
