# Supplementary material for: Clinical characteristics, antimicrobial resistance, and mortality of neonatal bloodstream infections in Northern Tanzania, 2022–2023
Source: PLoS One. 2025 Mar 25;20(3):e0319816. doi: 10.1371/journal.pone.0319816 (PMC11936297; doi:10.1371/journal.pone.0319816)
Supplement: S3 Table — (DOCX) [file pone.0319816.s003.docx]

**Supplementary Table 3: Characteristics of study participants with hospital-onset BSI, Kilimanjaro Christian Medical Centre, Tanzania, 2022-23**

| **Characteristics** | | **Infants with  HO-BSI**  (N = 17) | | **Infants with  non-HO-BSI**  (N = 89) | | **p-value** |
| --- | --- | --- | --- | --- | --- | --- |
| Female sex, n (%) | | 6 | (35.3) | 53 | (59.6) | 0.90 |
| Gestation age, n (%) | |  | | | | |
|  | Term (≥ 38 weeks) | 2 | (11.8) | 45 | (50.6) | **0.006** |
|  | Moderate-late preterm (32-37 weeks) | 9 | (52.9) | 26 | (29.2) |  |
|  | Very preterm (28-32 weeks) | 3 | (17.6) | 15 | (16.9) |  |
|  | Extremely preterm (< 28 weeks) | 3 | (17.6) | 3 | (3.4) |  |
| Birthweight < 2500 grams, n (%) | | 15 | (88.2) | 46 | (51.7) | **0.012** |
| Birthweight, mean (SD), grams | | 1568 | (709) | 2406 | (972) | **0.001** |
| Birthplace, n (%) | | | | | |  |
|  | KCMC | 4 | (23.5) | 35 | (39.3) | 0.34 |
|  | Home | 3 | (17.6) | 8 | (9.0) |  |
|  | Other hospital | 10 | (58.8) | 46 | (51.7) |  |
| Vaginal delivery, n (%) | | 11 | (64.7) | 55 | (61.8) | 1.00 |
| Maternal age, median (IQR), years | | 26 | (22, 31) | 28 | (23, 33) | 0.52 |
| Number of prior pregnancies, n (%)^1^ | | | | | |  |
|  | 0 | 3 | (17.6) | 24 | (27.0) | 0.80 |
|  | 1 | 5 | (29.4) | 29 | (32.6) |  |
|  | 2 | 5 | (29.4) | 10 | (11.2) |  |
|  | 3+ | 4 | (23.5) | 25 | (28.0) |  |
| Number of doses of maternal malaria prophylaxis received, n (%) | | | | | |  |
|  | 0 | 2 | (11.8) | 14 | (15.7) | — |
|  | 1-2 | 13 | (47.1) | 32 | (36.0) |  |
|  | 3-4 | 2 | (11.8) | 43 | (48.3) |  |
| Maternal HIV infection, n (%) | | 1 | (5.9) | 2 | (2.2) | 0.98 |
| Maternal antibacterials during pregnancy, n (%) | | 3 | (17.6) | 26 | (29.2) | 0.49 |
| Maternal antibacterials during delivery, n (%) | | 8 | (47.1) | 38 | (42.7) | 0.95 |
| Prolonged rupture of membranes ≥18 hours, n (%)^2^ | | 4 | (23.5) | 18 | (20.2) | 0.87 |
| Respiratory support on admission, n (%) | | 13 | (76.5) | 61 | (68.5) | 0.72 |
| Receipt of antibacterials prior to study enrollment, n (%) | | 17 | (100.0) | 83 | (93.3) | 0.60 |
| Receipt of antibacterials during admission, n (%) | | 17 | (100.0) | 88 | (98.9) | 1.00 |
| Length of stay, median (IQR), days | | 29.2 | (19.9, 38.5) | 8.3 | (6.7, 9.9) | **<0.001** |
| Died while hospitalized, n (%) | | 7 | (41.2) | 22 | (24.7) | 0.27 |
| Abbreviations: hospital-onset BSI (HO-BSI), bloodstream infection (BSI), day of life (DOL), interquartile range (IQR), 95% confidence interval (CI)  ^1^ Prior pregnancy data was missing from one infant with non-HO-BSI. ^2^ Prolonged rupture of membranes > 18 hours data was missing from one infant with non-HO-BSI. Statistical significance was evaluated at p-values of <0.05, statistically significant values are bolded. All dashes mean not applicable. | | | | | | |
