# Supplementary material for: Clinical characteristics, antimicrobial resistance, and mortality of neonatal bloodstream infections in Northern Tanzania, 2022–2023
Source: PLoS One. 2025 Mar 25;20(3):e0319816. doi: 10.1371/journal.pone.0319816 (PMC11936297; doi:10.1371/journal.pone.0319816)
Supplement: S4 Table — (DOCX) [file pone.0319816.s004.docx]

**Supplementary Table 4: Adjudication of ineffective antibacterial therapy among participants with bloodstream infection, Kilimanjaro Christian Medical Centre, Tanzania, 2022-23**

| **Organism isolated** | **Inpatient antibacterials prescribed** | | | | | | | | **Reason for  ineffective therapy^*^** | | | |
| --- | --- | --- | --- | --- | --- | --- | --- | --- | --- | --- | --- | --- |
|  |  |  |  |  |  |  |  |  | **1** | **2** | **3** | **4** |
|  | **Amp-Clox** | **Gent** | **AXO** | **Mero** | **Pip-tazo** | **Vanc** | **Cipro** | **Metro** |  |  |  |  |
| **Participant died while hospitalized (n = 8)** | | | | | | | | | | | | |
| Yeast |  |  |  |  |  |  |  |  | x |  |  |  |
| Yeast |  |  |  |  |  |  |  |  | x |  |  |  |
| *Yeast subtotal:* | | | | | | | | | *2* | *0* | *0* | *0* |
| *K. pneumoniae* |  |  |  |  |  |  |  |  | x |  |  |  |
| *K. pneumoniae* |  |  |  |  |  |  |  |  |  |  | x |  |
| *K. pneumoniae* |  |  |  |  |  |  |  |  |  |  | x |  |
| *A. baumanii* |  |  |  |  |  |  |  |  |  |  | x |  |
| *Gram-negative bacteria subtotal:* | | | | | | | | | *1* | *0* | *3* | *0* |
| CoNS |  |  |  |  |  |  |  |  |  |  | x |  |
| CoNS |  |  |  |  |  |  |  |  |  |  |  | x |
| *Gram-positive bacterial subtotal:* | | | | | | | | | *0* | *0* | *1* | *1* |
| **Participant discharged or remained alive, admitted at 28 day follow up (n = 42)** | | | | | | | | | | | | |
| Yeast |  |  |  |  |  |  |  |  | x |  |  |  |
| Yeast |  |  |  |  |  |  |  |  | x |  |  |  |
| Yeast |  |  |  |  |  |  |  |  | x |  |  |  |
| Yeast |  |  |  |  |  |  |  |  | x |  |  |  |
| Yeast |  |  |  |  |  |  |  |  | x |  |  |  |
| Yeast |  |  |  |  |  |  |  |  | x |  |  |  |
| *Yeast subtotal:* | | | | | | | | | *6* | *0* | *0* | *0* |
| *K. pneumoniae* |  |  |  |  |  |  |  |  |  | x |  |  |
| *K. pneumoniae* |  |  |  |  |  |  |  |  |  |  | x |  |
| *K. pneumoniae* |  |  |  |  |  |  |  |  |  |  | x |  |
| *K. pneumoniae* |  |  |  |  |  |  |  |  |  |  | x |  |
| *K. pneumoniae* |  |  |  |  |  |  |  |  |  |  | x |  |
| *K. pneumoniae* |  |  |  |  |  |  |  |  |  |  | x |  |
| *K. pneumoniae* |  |  |  |  |  |  |  |  |  |  | x |  |
| *K. pneumoniae* |  |  |  |  |  |  |  |  |  |  |  | x |
| *K. pneumoniae* |  |  |  |  |  |  |  |  |  |  |  | x |
| *Gram-negative bacteria subtotal:* | | | | | | | | | *0* | *1* | *6* | *2* |
| *S. aureus*, methicillin-sensitive |  |  |  |  |  |  |  |  |  |  |  | x |
| *S. aureus*, methicillin-sensitive |  |  |  |  |  |  |  |  |  |  |  | x |
| *S. aureus*, methicillin-resistant |  |  |  |  |  |  |  |  | x |  |  |  |
| CoNS |  |  |  |  |  |  |  |  | x |  |  |  |
| CoNS |  |  |  |  |  |  |  |  | x |  |  |  |
| CoNS |  |  |  |  |  |  |  |  | x |  |  |  |
| CoNS |  |  |  |  |  |  |  |  | x |  |  |  |
| CoNS |  |  |  |  |  |  |  |  | x |  |  |  |
| CoNS |  |  |  |  |  |  |  |  |  |  | x |  |
| CoNS |  |  |  |  |  |  |  |  |  |  | x |  |
| CoNS |  |  |  |  |  |  |  |  |  |  | x |  |
| CoNS |  |  |  |  |  |  |  |  |  |  | x |  |
| CoNS |  |  |  |  |  |  |  |  |  |  | x |  |
| CoNS |  |  |  |  |  |  |  |  |  |  |  | x |
| CoNS |  |  |  |  |  |  |  |  |  |  |  | x |
| CoNS |  |  |  |  |  |  |  |  |  |  |  | x |
| CoNS |  |  |  |  |  |  |  |  |  |  |  | x |
| CoNS |  |  |  |  |  |  |  |  |  |  |  | x |
| CoNS |  |  |  |  |  |  |  |  |  |  |  | x |
| CoNS |  |  |  |  |  |  |  |  |  |  |  | x |
| CoNS |  |  |  |  |  |  |  |  |  |  |  | x |
| CoNS |  |  |  |  |  |  |  |  |  |  |  | x |
| CoNS |  |  |  |  |  |  |  |  |  |  |  | x |
| CoNS |  |  |  |  |  |  |  |  |  |  |  | x |
| CoNS |  |  |  |  |  |  |  |  |  |  |  | x |
| CoNS |  |  |  |  |  |  |  |  |  |  |  | x |
| CoNS |  |  |  |  |  |  |  |  |  |  |  | x |
| *Gram-positive bacteria subtotal:* | | | | | | | | | *6* | *0* | *5* | *16* |
| **Totals:** | | | | | | | | | **15** | **1** | **15** | **19** |
| Abbreviations: ampicillin-cloxacillin (amp-clox), gentamicin (gent), ceftriaxone (AXO), meropenem (mero), piperacillin-tazobactam (pip-tazo), vancomycin (vanc), ciprofloxacin (cipro), metronidazole (metro), coagulase-negative *Staphylococcus* species (CoNS).  Grayed box indicates antibacterial was prescribed.  ^*^ Ineffective therapy was defined and coded as one or more the following:   1. Participant was prescribed an antibacterial without adequate activity against the identified organism or intrinsic resistance 2. No antimicrobial was prescribed to the participant 3. The identified organism generally has susceptibility to the prescribed antibacterial, but the specific isolate demonstrated   resistance against the antibacterial the participant was prescribed 4. The participant was prescribed effective antibacterial(s) for fewer than 7 days of therapy | | | | | | | | | | | | |
